# Locally Confined Clonal Complexes of *Mycobacterium ulcerans* in Two Buruli Ulcer Endemic Regions of Cameroon

**DOI:** 10.1371/journal.pntd.0003802

**Published:** 2015-06-05

**Authors:** Miriam Bolz, Martin W. Bratschi, Sarah Kerber, Jacques C. Minyem, Alphonse Um Boock, Moritz Vogel, Pierre Franklin Bayi, Thomas Junghanss, Daniela Brites, Simon R. Harris, Julian Parkhill, Gerd Pluschke, Araceli Lamelas Cabello

**Affiliations:** 1 Swiss Tropical and Public Health Institute, Basel, Switzerland; 2 University of Basel, Basel, Switzerland; 3 FAIRMED, Yaoundé, Cameroon; 4 Section Clinical Tropical Medicine, Heidelberg University Hospital, Heidelberg, Germany; 5 Wellcome Trust Sanger Institute, Wellcome Trust Genome Campus, Hinxton, Cambridge, United Kingdom; Fondation Raoul Follereau, FRANCE

## Abstract

**Background:**

*Mycobacterium ulcerans* is the causative agent of the necrotizing skin disease Buruli ulcer (BU), which has been reported from over 30 countries worldwide. The majority of notified patients come from West African countries, such as Côte d’Ivoire, Ghana, Benin and Cameroon. All clinical isolates of *M*. *ulcerans* from these countries are closely related and their genomes differ only in a limited number of single nucleotide polymorphisms (SNPs).

**Methodology/Principal Findings:**

We performed a molecular epidemiological study with clinical isolates from patients from two distinct BU endemic regions of Cameroon, the Nyong and the Mapé river basins. Whole genome sequencing of the *M*. *ulcerans* strains from these two BU endemic areas revealed the presence of two phylogenetically distinct clonal complexes. The strains from the Nyong river basin were genetically more diverse and less closely related to the *M*. *ulcerans* strain circulating in Ghana and Benin than the strains causing BU in the Mapé river basin.

**Conclusions:**

Our comparative genomic analysis revealed that *M*. *ulcerans* clones diversify locally by the accumulation of SNPs. Case isolates coming from more recently emerging BU endemic areas, such as the Mapé river basin, may be less diverse than populations from longer standing disease foci, such as the Nyong river basin. Exchange of strains between distinct endemic areas seems to be rare and local clonal complexes can be easily distinguished by whole genome sequencing.

## Author Summary

Buruli ulcer (BU) is a progressively necrotizing disease of the skin, caused by infection with *Mycobacterium ulcerans*. BU occurs very focally with highest incidence in West Africa. The mode of transmission and the nature and role of potential environmental reservoirs are currently not entirely understood. In this study we sequenced whole genomes of sets of *M*. *ulcerans* case isolates from two BU endemic regions in Cameroon. We identified two distinct phylogenetic lineages, which directly correlated with the two endemic regions. Furthermore, we showed that the genetic diversity of *M*. *ulcerans* is higher in the older endemic region of Cameroon (Nyong river basin) compared to the more recently emerged infection focus in the same country (Mapé river basin). Together, our results demonstrate that *M*. *ulcerans* is developing local clonal complexes by the accumulation of single nucleotide polymorphisms (SNPs) and that these complexes often remain confined to individual endemic foci. The gene encoding for rpoB, which is known to harbour drug resistance mutations against rifampicin in *M*. *tuberculosis*, was not affected by SNPs in any of the analysed *M*. *ulcerans* strains.

## Introduction

Buruli ulcer (BU), the third most common mycobacterial disease affecting humans after tuberculosis and leprosy, is caused by *Mycobacterium ulcerans* [1]. The disease is characterized by progressive necrosis of the skin and subcutaneous tissue, leading to sometimes extensive ulcerations. Even though standard antibiotic treatment for eight weeks with rifampicin and streptomycin, as it is currently recommended by the World Health Organization (WHO), is highly effective in killing the bacterium, quite a number of patients still require surgery for wound debridement and/or skin grafting and can remain with scarring and disabilities [2]. BU has been reported from over 30 countries worldwide but has its highest incidence in West Africa, where it occurs very focally in rural areas, which are associated with wetlands, marshes and riverine zones [3].

The distinct pathology of *M*. *ulcerans* infections is mainly attributed to mycolactone, a macrolide exotoxin produced by the mycobacteria [4]. Mycolactone is highly toxic to mammalian cells and is also believed to have immunomodulatory functions [5]. The polyketide synthases required to produce this potent toxin are encoded as three large genes on a giant virulence plasmid, pMUM001, whose acquisition represents a crucial step in the divergence of *M*. *ulcerans* from its progenitor *M*. *marinum* [6,7]. Further hallmarks of *M*. *ulcerans* evolution include the proliferation of two distinct insertion sequence (IS) elements (IS2404 and IS2606), the accumulation of a large number of pseudogenes and considerable genome downsizing. These findings indicate that *M*. *ulcerans* has recently passed through an evolutionary bottleneck and is adapting to a new and more stable environment [7]. This new niche is suspected to be aerobic, osmotically stable, dark and possibly extracellular. Production of the immunosuppressive toxin mycolactone and the loss of a set of highly immunogenic proteins [8] may represent an adaptation to an environment that is screened by an immune system.

Possums, an Australian marsupial species, seem to be especially susceptible to the disease and may function as an animal reservoir in BU endemic foci of Victoria, Australia. However, attempts to identify an animal reservoir in Africa have not been successful to date [9,10]. Therefore it is assumed that there may be other environmental reservoirs of *M*. *ulcerans* in association with stagnant water bodies. Contact with such wetlands is a known risk factor for contracting BU, and patients may become infected through microtrauma of the skin or inoculation by an unknown insect vector [3]. *M*. *ulcerans* DNA has been detected by IS2404 specific PCR in environmental samples, but the cultivation of the slow growing mycobacteria from such samples is exceptionally difficult and has only succeeded once so far [11,12]. Therefore it remains unclear what relevance the presence of DNA in the environment has and how *M*. *ulcerans* is transmitted. For a long time the highly clonal population structure of *M*. *ulcerans* represented a major obstacle for molecular epidemiological studies. Conventional typing methods such as restriction fragment length polymorphism, multilocus sequence typing and variable number tandem repeat analysis provide insufficient resolution [13]. To date the best typing resolution was attained with single nucleotide polymorphism (SNP) typing assays [14]. With this method *Röltgen et al*. were able to demonstrate focal transmission in the Densu river basin of Ghana [14]. Furthermore, with an extended set of SNPs sufficient phylogenetic signal could be obtained to reconstruct recent evolutionary events in *M*. *ulcerans* on a continental scale [15]. A drawback of this method is, however, that it requires prior knowledge of the relevant SNPs.

With its decreasing costs, whole genome sequencing (WGS) is now replacing SNP typing for *M*. *ulcerans*. Here we report on a genomic epidemiological study aimed at inferring evolutionary patterns of *M*. *ulcerans* in two BU endemic regions of Cameroon (the Mapé and the Nyong river basins) by using WGS combined with fine-scale geographic information on the origin of the patients from which the *M*. *ulcerans* strains were isolated.

## Materials and Methods

### Ethical statement and patient recruitment

Samples for this study were collected from patients recruited between August 2010 and July 2012 in the Mapé river basin of Cameroon [16] and at the district hospital in Ayos in southern Cameroon [17]. Ethical clearance for the collection and processing of samples was obtained from the Cameroon National Ethics Committee (N°041/CNE/DNM/09, N°006/CNE/SE/2010, and N°172/CNE/SE/2011), the Ethics Committee of the Heidelberg University Hospital, Germany (N°ISRCTN72102977) and the Ethics Committee of Basel (EKBB, reference n. 53/11). Participation was voluntary and all patients who participated in the study or their legal guardian provided written informed consent.

### *M*. *ulcerans* cultivation

Prior to the start of medical treatment, cotton swabs were collected from each patient for diagnosis of *M*. *ulcerans* disease by quantitative polymerase chain reaction (qPCR) targeting the *M*. *ulcerans* specific IS2404 [18] and for cultivation of the bacteria. Wound exudates in phosphate buffered saline (PBS), that were produced from cotton swabs for DNA extraction as described by Lavender and Fyfe [19], were decontaminated as described by *Bratschi et al*. [20] and cultures initiated on Löwenstein-Jensen (LJ) medium slants (with glycerol; Becton Dickinson and Company) and/or LJ medium slants supplemented with 2% PANTA. Inoculated cultures were incubated at 30°C until growth could be observed. Detected growth was confirmed to be *M*. *ulcerans* by colony PCR using primers MU154 (5’-ggcagttacttcactgcaca-3’) and MU155 (5’-cggtgatcaagcgttcacga-3’) and amplification for 32 cycles of 30 seconds at 94°C, 30 seconds at 60°C and 1 minute at 72°C. PCR products were resolved in a 1.5% agarose gel. Confirmed *M*. *ulcerans* cultures were expanded on 7H10 agar plates (Becton Dickinson and Company) until enough bacteria could be harvested for DNA extraction as described below.

### DNA extraction

*M*. *ulcerans* DNA for WGS was extracted as described by *Käser et al*. [21]. Briefly, *M*. *ulcerans* bacteria were transferred from 7H10 agar plates into a 1.5ml screw-cap tube and suspended in lysis buffer (15% sucrose, 50 mM Tris (pH8.5) and 50 mM EDTA). After Incubation with lysozyme for 1h at 37°C, sodium dodecyl sulfate (SDS) and proteinase K (PK) were added and the bacteria lysed with a tissue homogenizer (Precellys24, Bertin Technologies) in tough micro-organism lysis tubes containing beads (Bertin Technologies). DNA from lysate supernatant was extracted by the Phenol-chlorophorm / Ethyl alcohol (EtOH) method [21]. Amount and purity of the extracted mycobacterial DNA was assessed with a Qubit 2.0 Fluorometer according to the manufacturer’s protocol (Qubit dsDNA HS Assay Kit, Invitrogen).

### Whole genome sequencing

All processing and sequencing of genomic DNA was performed by the core sequencing teams at the Wellcome Trust Sanger Institute. All samples were sequenced as multiplexed libraries using Illumina HiSeq 2000 analyzers on 75-bp paired-end runs as described by *Harris et al*. [22].

### Read alignment and SNP detection

Variation, in the form of SNPs, was detected using a mapping approach. The paired-end Illumina reads were mapped against the *M*. *ulcerans* reference genome of the strain Agy99 (accession number CP000325) and against the *M*. *ulcerans* Agy99 pMUM001 plasmid, with an insert size ranging from 50 to 400 bp using SMALT version 0.7.4 (http://www.sanger.ac.uk/resources/software/smalt/) with a word length of 13 and skip size of 1. The maximum insert size was 1000, the minimum insert size was 50, resulting, on average, in a 25x depth coverage for more than 92.1% of the reference genome. The default mapping parameters recommended for reads were employed, except for the minimum score required for mapping, which was increased to 30 to make the mapping more conservative. Candidate SNPs were identified using SAMtools [23] mpileup as previously described [24]. Base calls for all isolates were filtered to remove those at sites with a SNP quality score below 30, where the called based was in less than 75% of mapped reads on each strand, or where fewer than two reads mapped to each strand. SNPs called in repetitive regions of the *M*. *ulcerans* reference genome (737,280 bp) were excluded from the analysis and only the SNPs mapped in the core genome (4,894,326 bp) were used to construct the phylogenetic tree. Repetitive regions were defined as exact repetitive sequences of ≥20 bp in length, identified using rep repeat-match [25]. If 10% of the genomes under study had an ambiguous base in a called SNP, these positions were removed from the analysis. The information on the potentially problematic regions is provided in S1 Table.

### Phylogenetic analyses

A maximum-likelihood phylogenetic tree was generated from the whole genome sequences based on the SNPs called by SMALT. Published *M*. *ulcerans* genomes from strains isolated in Ghana (NM14_01, NM49_02, NM54_02, NM43_02, Agy99), Benin (Mu_06–3845, Mu_06–3846, Mu_07–1082) and the *M*. *marinum* genome (Mu_06–3844) of an isolate from Belgium were included in the analysis [13]. The *M*. *marinum* genome was used to root the tree. In total 91 strains were included in this study (S2 Table). Separate maximum-likelihood trees for the plasmid and the chromosome were reconstructed based on one *M*. *marinum* genome and 53 *M*. *ulcerans* genomes. The *M*. *ulcerans* genomes include 45 Cameroonian isolates with each patient being represented by one strain. Maximum-likelihood phylogenetic trees were constructed using RAxML v7.0.4 und a general time-reversible (GTR) substitution model with γ correction for among-site rate variation. Support for nodes on the trees was assessed using 100 bootstrap replicates.

### SNP pairwise distance

To compute the pairwise distance based on the genome wide SNP count between the isolates we used MEGA6 [26]. The average pairwise SNP distances per genome within the lineages were plotted using software package R (http://www.r-project.org/) and statistical significance was assessed by applying the Wilcoxon rank sum test.

### Nucleotide diversity

Genetic diversity was assessed by calculating the average pair-wise nucleotide differences per site (Pi) for both main lineages of *M*. *ulcerans* in Cameroon, using the program VariScan [27]. We calculated the nucleotide diversity for 1.5 kb non-overlapping windows.

### Median joining networks

We produced two median joining networks using Network 4.6.1.2 [28], based on 107 variable nucleotide positions from 34 isolates of the Mapé river basin and 117 variable nucleotide positions from 11 isolates of the Nyong river basin. For the analysis of the association between geographic and genetic distances of the sampled populations we performed a Mantel test using the function “mantel” asking for the Pearson’s product –moment correlation with 999 permutations in the R package “vegan” [29]. We did this for two sets of matrices, a smaller subset of 11 samples corresponding to the Nyong isolates, and the entire set of 33 samples corresponding to the Mapé isolates.

## Results

### *M*. *ulcerans* phylogeny in Cameroon

We sequenced the genomes of 82 *M*. *ulcerans* strains isolated from 45 IS2404 qPCR confirmed Cameroonian BU patients. Patients were identified between 2010 and 2012 and came from two geographically separated BU endemic regions of Cameroon, the Mapé and the Nyong river basins. Prior to treatment, ulcerative lesions were sampled with a cotton swab for laboratory confirmation of the clinical diagnosis, primary isolation of the disease causing organism and WGS of the isolated *M*. *ulcerans* strains.

**Fig 1:**
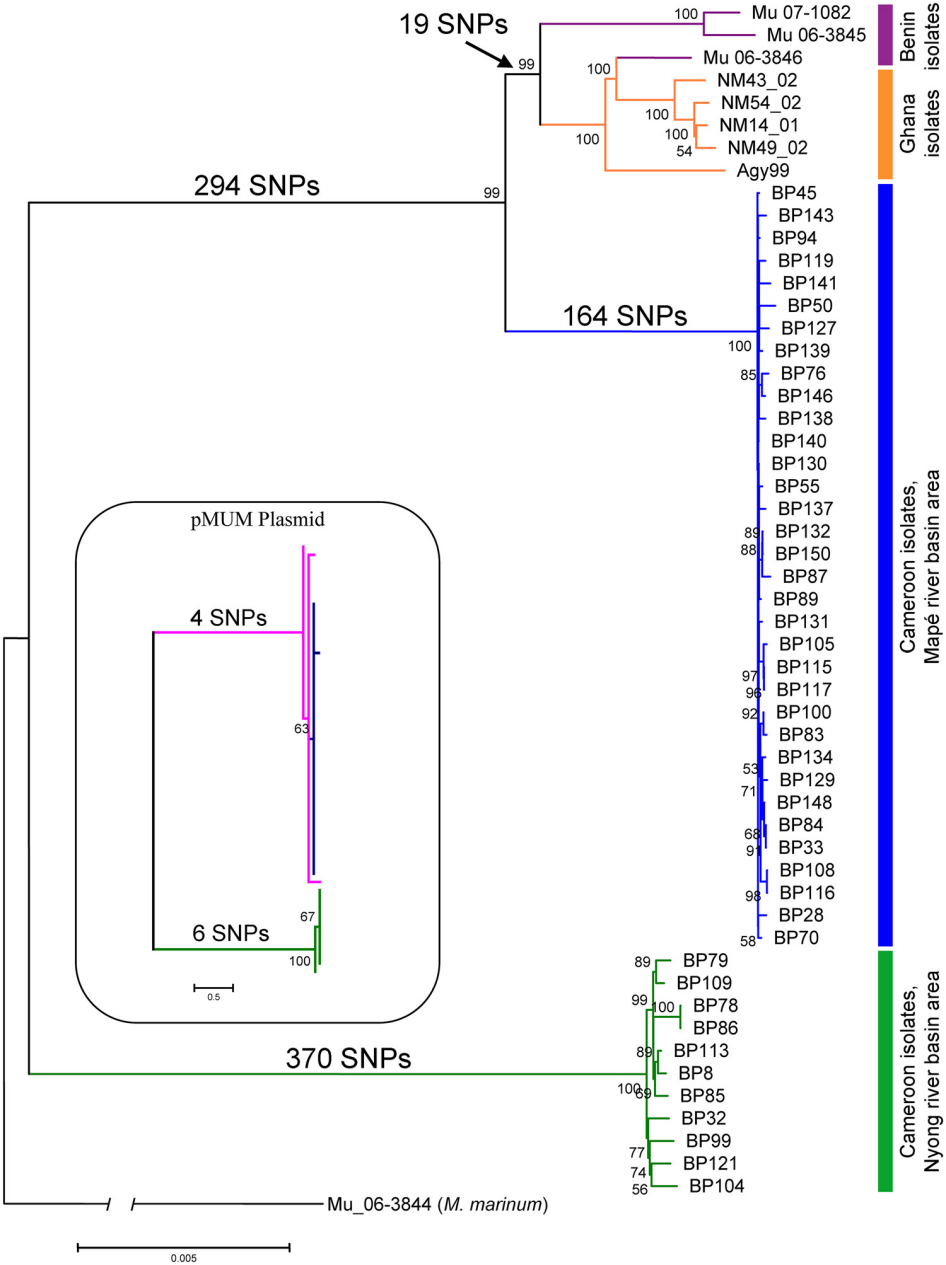
Phylogenetic reconstruction of African *M*. *ulcerans* strains. Maximum-likelihood phylogenetic tree based on 26,740 variable nucleotide positions across 53 *M*. *ulcerans* strains by RAxML. The tree was rooted using *M*. *marinum* (Mu_06–3844) as outgroup. The geographical origin of the strains is indicated to the right of the tree and branches are coloured according to the region of isolation of the strains. Bootstrap values higher than 50% are shown along the branches. The insert shows the pMUM plasmid SNP tree based on 21 SNPs with the topology matching the corresponding chromosome-based tree.

The mapping of the obtained Illumina sequencing reads against the reference strain resulted in an average coverage of 380 reads per position per genome. We reconstructed the phylogenetic relationship among 45 Cameroonian (one strain per patient), five Ghanaian and three Beninese *M*. *ulcerans* isolates based on 26,740 variable nucleotide positions, rooting the tree using a published *M*. *marinum* genome (Fig 1). The phylogenetic tree showed a very strong geographical structure. The chromosome tree of the Cameroonian *M*. *ulcerans* isolates showed two distinct lineages, the first one containing all the Nyong river basin isolates (Nyong lineage) and the second one all the isolates from the Mapé region (Mapé lineage). The Mapé river basin isolates were more closely related to a set of published genomes [13] of Ghanaian and Beninese isolates that we included in the analysis, than to the Nyong lineage. The two Cameroonian clonal complexes differed in altogether 828 SNPs shared by all members of the respective lineages (Fig 1).

The plasmid phylogeny reflected the topology of the *M*. *ulcerans* chromosome phylogenetic tree (Fig 1), supporting the hypothesis of a unique acquisition of the plasmid during the emergence of *M*. *ulcerans* [30] followed by parallel evolution between the chromosome and the plasmid.

### Genetic diversity among the Cameroonian isolates

**Fig 2:**
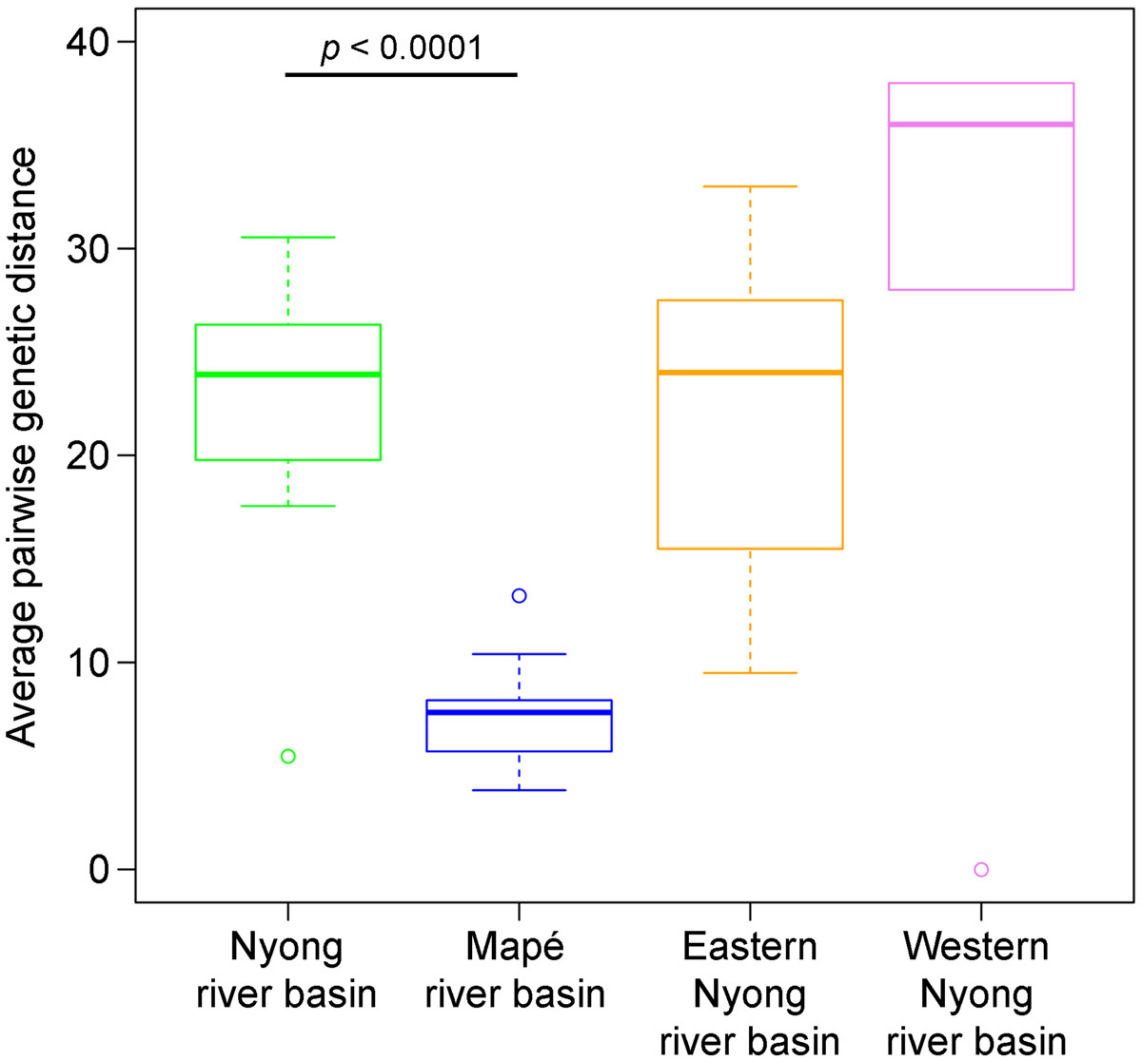
SNP pairwise distance between isolates from the same lineage. The variation in the average SNP pairwise distance per genome for each lineage (Mapé and Nyong river basin strains) is shown in a box plot, with circles representing outlier sequence pairs. Two sub-areas of the Nyong river basin: the Eastern Nyong river basin and Western Nyong river basin, which are each smaller than the Mapé river basin area, are additionally shown. Statistical significance was assessed with the Wilcoxon rank sum test.

**Fig 3:**
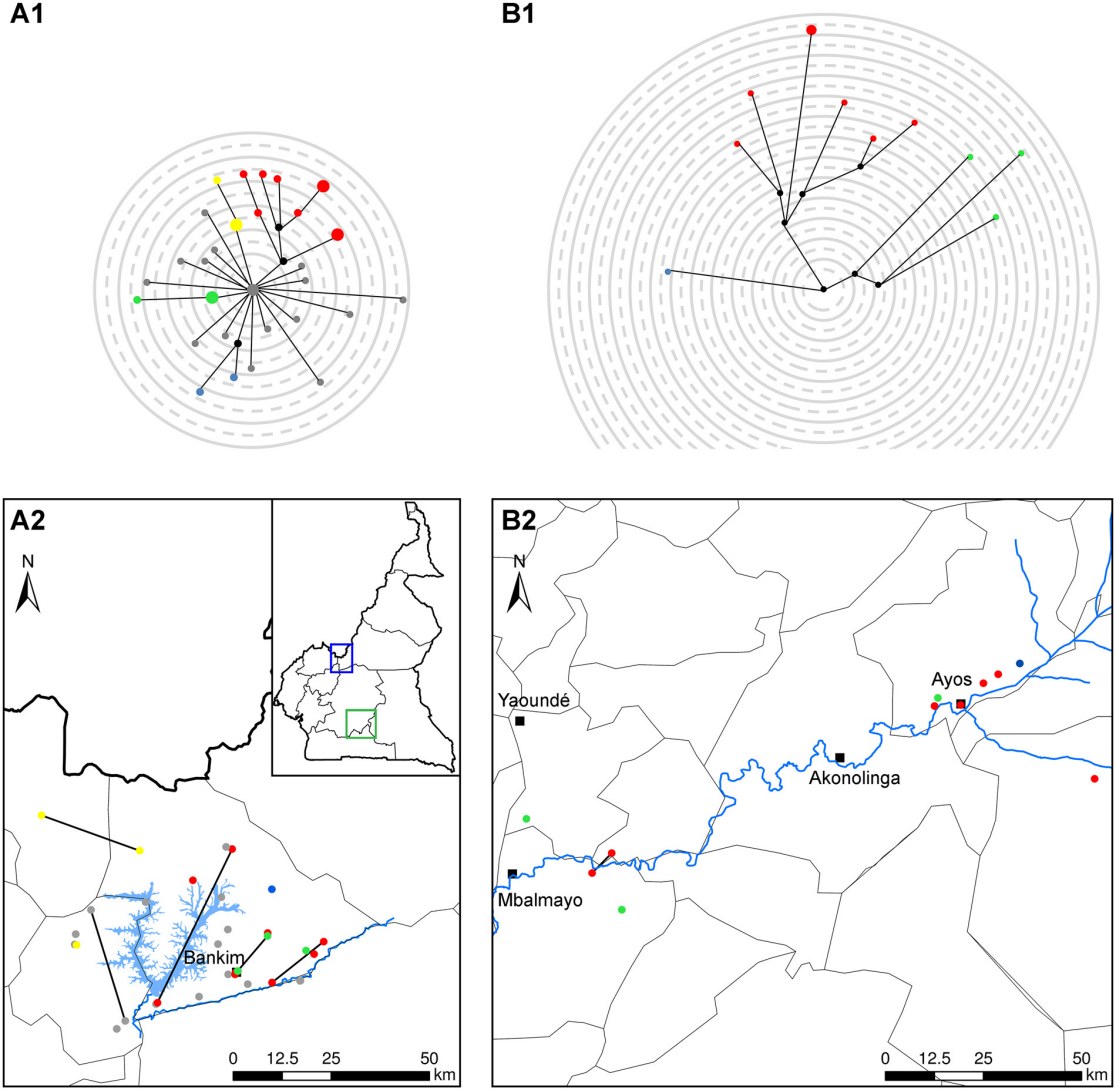
Phylogeographic analysis of *M*. *ulcerans* in the Mapé and Nyong river basins of Cameroon. Detailed information about the places of residence of the patients for the year before the onset of BU disease was collected from all patients from whom *M*. *ulcerans* was isolated. Micro-evolutionary diversity of *M*. *ulcerans* at the two BU endemic sites is shown in panel A1 (Mapé river basin) and panel B1 (Nyong river basin). Median joining networks using 117 variable single nucleotide positions among whole genome sequences of *M*. *ulcerans* isolates from the Mapé river basin and 107 positions in isolates from the Nyong river basin area are depicted. Branch lengths correspond to the number of SNP differences, circle sizes correspond to the number of isolates and circle colours correspond to the sub-clusters defined. Black circles indicate median vectors (mv) and are hypothetical genotypes. Panels A2 (Mapé river basin) and B2 (Nyong river basin) show the geographic localization of the patient’s residences with the locations colour coded according to the lineages shown in panel A1 and B1. When isolates of two patients were exactly the same, the respective homes of the patients are connected by a line. Major towns in the areas are indicated by black squares.

We analysed the genetic diversity within the two Cameroonian geographical lineages separately (Fig 2). The genetic diversity observed for the Nyong river basin isolates (median pairwise SNP difference = 26.2 SNPs) was significantly higher (*p*-value < 0.0001) than for the Mapé basin isolates (median pairwise SNP difference = 7.6 SNPs). Furthermore, an analysis of the pairwise geographic distance of seven isolates from the Eastern Nyong river basin (approximately 1090 km^2^) and of four isolates from Western Nyong (approximately 625km^2^) still yielded values that were higher (median 24 and 36 pairwise SNP difference) than for the Mapé isolates (Fig 2). The higher genetic diversity thus does not seem to be related to the broader geographical distribution for the Nyong river basin isolates (approximately 8600 km^2^) compared to the Mapé river basin isolates (approximately 6400 km^2^) (Fig 3A2 and 3B2). These results were also reflected in the phylogenetic tree, where branch lengths were longer for the Nyong river basin strains than for the Mapé river basin isolates (Fig 1). The gene encoding for rpoB, which is known to harbour drug resistance mutations against rifampicin in *M*. *tuberculosis* [31], was not affected by SNPs in any of the *M*. *ulcerans* strains analysed here.

When analysing the nucleotide diversity distribution along the chromosome by calculating the average nucleotide pairwise diversity per site (Pi) for both lineages, 99.9% of the genome was found to be highly conserved (S1 Fig). However, the average nucleotide diversity per site for the Nyong river basin lineage was 3.2 times higher than for the Mapé river basin lineage (4.10e^-6^ *versus* 1.3e^-6^). The regions of the genome with higher nucleotide diversity (0.375e^-4^ and 1.25e^-4^, respectively) seemed randomly distributed across the chromosome for both lineages (S2 Fig) and the gene content of these regions varied between the two Cameroonian lineages, comprising affected genes of diverse functionalities (S3 Table).

### Phylogeographic analysis of the Cameroonian *M*. *ulcerans* isolates

In order to analyse the distribution of genetic variants within the endemic areas, we reconstructed median joining networks for the sequenced strains and mapped the places of residence of the patients from which the strains originated (Fig 3).

The network of the Mapé river basin isolates had a star structure with two isolates (BP130 and BP140) at the centre. All the other isolates were connected to this centre and separated by three to nine SNPs. While the SNP distance between the two central strains was zero, the geographical distance between the corresponding residence places of the two corresponding patients was 19.5 km. A total of four clusters were distinguished in the network: blue formed by two isolates, green and yellow formed by three isolates each and the red cluster as a complex structure formed by nine strains. The strains belonging to the red cluster shared two SNPs, the green ones also two SNPs, the yellow ones four and the blue ones shared three SNPs. All the grey strains were not forming clusters and differed by 1 to 11 SNPs from the central strains. In the network of the Nyong river basin isolates we observed only three clusters formed by three (green), seven (red) and one strain (blue). The isolates from the red cluster shared six SNPs, while the isolates from the green one shared only two SNPs. Overall, for both BU endemic areas in Cameroon we did not find a clear correlation between the genetic networks and the geographic distribution of the houses where the patients lived in the year prior to the onset of BU disease (Fig 3). Statistical analysis with the Mantel test for the smaller subset of Nyong samples resulted in a positive and marginally significant correlation between the geographic and genetic distances (r = 0.2785, *p*-value = 0.054), whereas the test performed for the Mapé set of isolates resulted in a small non-significant negative correlation (r = -0.04774, *p*-value = 0.676).

### Rate of acquisition of SNPs

In the course of this genomic epidemiological study we obtained from three patients isolates from two or three different time points during the course of their disease (Table 1). When comparing the SNP diversity between the isolates from the same patient, only one SNP difference was observed between two sequential isolates (Table 1). The affected gene (MUL_1383) encoded for a hypothetical protein and the detected mutation was synonymous. No SNP difference was observed between two strains isolated from two distant ulcers of one patient (Table 1).

**Table 1:** Genomic diversity among strains isolated from consecutive swab samples from the same BU lesion and among isolates originating from different lesions of the same patient.

Patient ID	Target lesion	Sampling time (days of/after treatment) or sampling location	Swab ID	Big pellet ID	Number of SNPs compared to the first sampling time point or the other sampling location respectively
BU02_038	1	Day 89	MB2_0057	BP39	
		Day 232	MB2_0192	BP29	0
		Day 337	MB2_0373	BP58	0
BU02_51	1	Day 0	MB2_061	BP53	
		Day 128	MB2_0305	BP25	0
BU02_061	1	Day 0	MB2_0292	BP71	
		Day 25	MB2_0477	BP59	1
BU2011_49	1	shoulder	MB2_0385	BP48	
	2	ankle	MB2_0473	BP67	0

## Discussion

Due to the limitations of conventional typing methods for the differentiation of strains belonging to the highly monomorphic African *M*. *ulcerans* population, use of WGS was suggested to reach sufficient analytical depth for molecular epidemiology studies [32]. Here our comparative genomic analysis of strains from two geographically separated BU endemic areas of Cameroon, the Mapé and the Nyong river basins, identified two phylogenetically distinct lineages of *M*. *ulcerans*. These data support previous findings that the spread of local clonal lineages between endemic areas only rarely occurs [13,14]. In a previous IS element—SNP based typing study most strains from the central region of Cameroon had the same SNP types as strains from neighbouring Gabon. The IS element—SNP type found in a strain from the Mapé river basin was also present across entire Central and West-Africa leading to the hypothesis that this lineage represents the founder of the other observed IS element—SNP types [15]. Our WGS analysis showed that the strains from the Mapé river basin are in fact more closely related to the *M*. *ulcerans* strain circulating in Ghana and Benin than the strains belonging to the Nyong river basin lineage. Additional WGS data with strains from all BU endemic African countries are required to shed more light on the spread and evolution of *M*. *ulcerans* in Africa and the origin of the locally observed two distinct Cameroonian lineages.

Analysis of the pairwise SNPs distance and nucleotide diversity distribution revealed a lower genetic diversity among the Mapé river basin strains than among the Nyong river basin strains. Epidemiological data suggest that *M*. *ulcerans* has expanded in the Mapé river area more recently than in the Nyong river basin. Descriptions of BU cases in the Nyong river basin exist since 1969 [17]. In contrast, clinically suspected cases of BU in the Mapé river area have been reported first only in 2004 [33]. While the disease may have preexisted there, epidemiological data strongly indicate that BU incidence has recently increased in the Mapé river basin [16,33]. Recent expansion of a clone may thus explain the more limited genetic diversity of the *M*. *ulcerans* lineage present in the Mapé river basin. It was speculated, that this expansion was associated with environmental changes caused by the damming of the Mapé river in 1989 [16,33]. Although it has been shown that cases associate more with the Mbam river as opposed to the Mapé dam directly [11,16], damming may have had an indirect effect on groundwater level and stagnant water bodies in the area.

We have compared the genome sequences of strains isolated at different time points over the course of the BU infection of three patients. In only one case, we detected a single SNP in one of the isolates compared to the strain isolated earlier from the same patient. It is not possible to conclude whether this observed single polymorphism is related to a re-infection by a variant strain or to a point mutation that occurred either in the patient or during the *in vitro* cultivation. However, these data support the expectation of a low mutation rate in *M*. *ulcerans*.

Our analysis shows that WGS is an important tool for studying the local diversity and population structure of *M*. *ulcerans* in endemic areas and for resolving the evolutionary history of the pathogen. A combination of phylogenetic analysis with geographical information on the patient’s home at the time of disease onset did not reveal a clear distribution pattern of the genetic variants. This may in part be related to the limited resolution of the comparative genomic analysis performed here. Resolution of the WGS typing could be further increased by inclusion of repetitive regions of the genome and the virulence plasmid that we so far excluded from the analysis, such as the IS2404 and PE/PPE regions. On the other hand, our sero-epidemiological analyses have provided evidence that exposure to *M*. *ulcerans* does not primarily occur at the homes of patients [34], but may rather be associated with more peripheral environmental water contact sites. Furthermore, for patients from the Mapé river basin it has been found that many of them move over long distances (in some cases >15 km) from their homes towards the Mbam river for their farming activities [11,16]. For genomic epidemiology studies it may therefore be necessary to establish detailed individual movement and environmental water contact patterns to follow the spatial-temporal spread of genetic variants.

## Supporting Information

S1 Fig Distribution of chromosomal nucleotide diversity statistics. Pi calculated on non-overlapping 1,500 pair sliding windows. Plots were drawn using the R density function.
(PDF)

S2 Fig Nucleotide diversity (Pi) values along the length of the chromosome. Chromosomes are represented linearly, using the coordinate system of the respective reference genomes with 0 on the far left (and far right).
(PDF)

S1 Table Potentially problematic regions for SNP calling identified by repeat-match. (XLSX)

S2 Table Mapping statistics and metadata for the bacterial isolates used in the study. (XLSX)

S3 Table Areas across the genomes with high Pi. (XLSX)
