# Supplementary figures and images for: Locally Confined Clonal Complexes of Mycobacterium ulcerans in Two Buruli Ulcer Endemic Regions of Cameroon
Source: PLoS Negl Trop Dis. 2015 Jun 5;9(6):e0003802. doi: 10.1371/journal.pntd.0003802 (PMC4457821; doi:10.1371/journal.pntd.0003802)

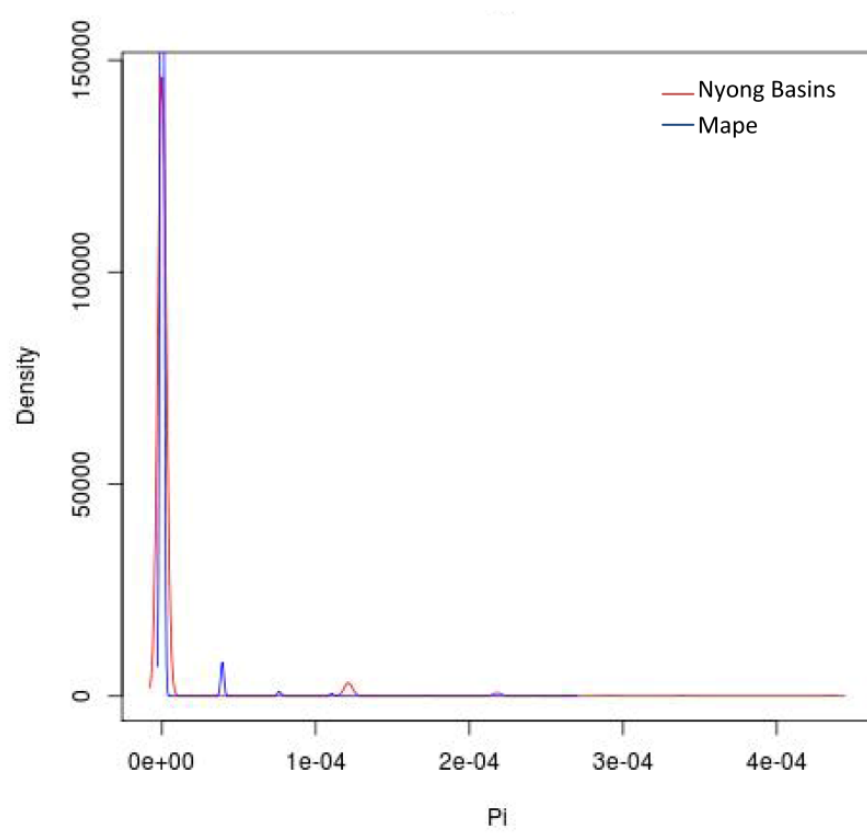

Supplement: S1 Fig — Pi calculated on non-overlapping 1,500 pair sliding windows. Plots were drawn using the R density function. (PDF) [file pntd.0003802.s001.pdf]

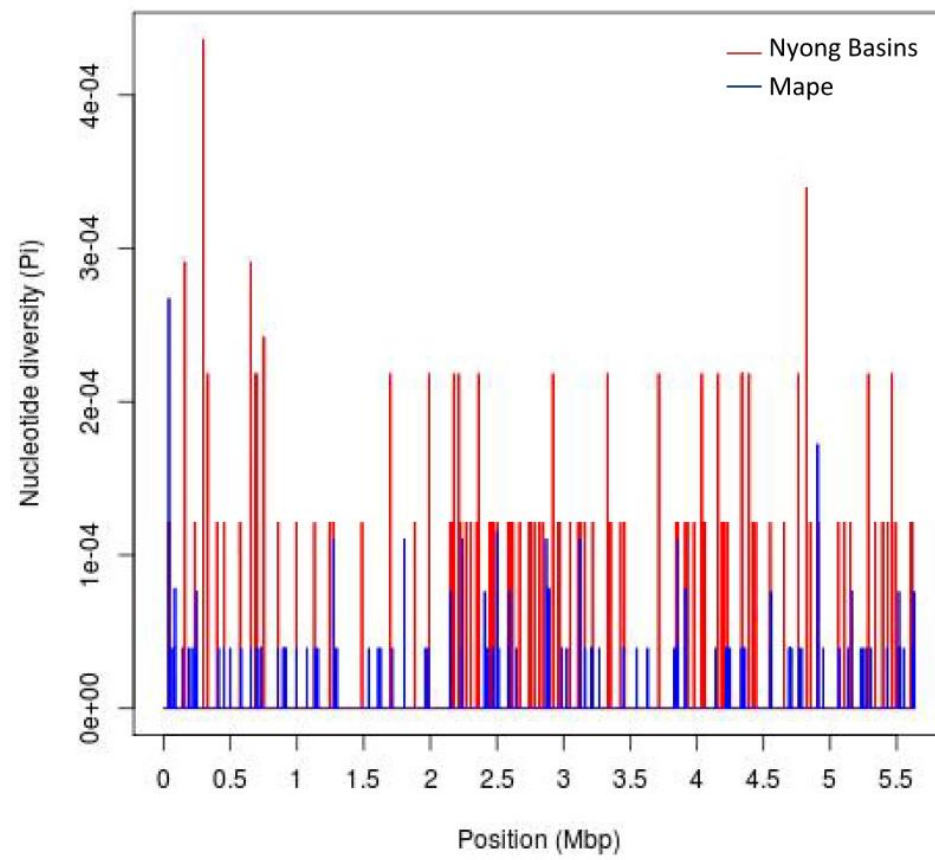

Supplement: S2 Fig — Chromosomes are represented linearly, using the coordinate system of the respective reference genomes with 0 on the far left (and far right). (PDF) [file pntd.0003802.s002.pdf]
